# Two-dimensional ultrasound signs as predictive markers of massive peri-operative blood loss in placenta previa suspicious for placenta accreta spectrum (PAS) disorder

**DOI:** 10.1371/journal.pone.0276153

**Published:** 2022-10-14

**Authors:** Wattanan Watthanasathitnukun, Savitree Pranpanus, Chusana Petpichetchian

**Affiliations:** Department of Obstetrics and Gynecology, Faculty of Medicine, Prince of Songkla University, Hat Yai, Songkhla, Thailand; Universita degli Studi dell’Insubria, ITALY

## Abstract

**Objective:**

To evaluate certain two-dimensional (2D) ultrasound signs as predictors of massive peri-operative blood loss (PBL) in pregnant women with placenta previa suspicious of PAS disorder.

**Materials and methods:**

A single center retrospective study was done in pregnant women who had undergone prenatal diagnosis with 2D ultrasound grey scale and color Doppler using the EW-AIP (which has been changed to IS-PAS (International Society for the Placenta Accreta Spectrum)) criteria between January 2007 and May 2021. The patients were divided into 2 groups, non-massive hemorrhage with PBL ≤ 2500 mL and massive PBL >2500 mL. All PAS cases had pathological confirmation. Ultrasound signs and hemorrhagic outcomes were compared between the two groups. A PAS scoring system to predict massive PBL was constructed and a receiver operating characteristic (ROC) curve was calculated to evaluate the efficacy of the scoring system.

**Results:**

Of 534 women, 146 (28.3%) had PBL > 2500 mL and 388 (71.7%) had PBL ≤ 2500 mL. In the massive PBL group, 101 (69.2%) were diagnosed as PAS and 45 (30.8%) as placenta previa alone. From 10 evaluated 2D ultrasound signs, 3 had the highest odds ratios (ORs) associated with massive PBL, ‘focal exophytic mass’ OR 8.17 (*p* = 0.024), ‘placental bulge’ OR 2.47 (*p* = 0.011), and ‘placental lacunae feeder vessels’ OR 2.38 (*p* = 0.01). When using the PAS scoring system, the AUC to predict massive PBL was 0.80 (95% CI, 0.76–0.85,).

**Conclusion:**

Our PAS scoring system based on 2-dimensional ultrasound signs combined with grey scale and color Doppler is useful to predict massive PBL and can help optimize pre-operative management in cases of previa suspicious of PAS.

## Introduction

Placenta accreta spectrum (PAS) disorder is a condition in which trophoblasts invade abnormally into the myometrium. PAS severity is classified into 3 groups based on the depth of invasion from histology, placenta accreta, placenta increta and placenta percreta [1]. The subgroups of PAS which can cause life-threatening hemorrhage are placenta increta and percreta, which are defined as abnormally invasive placenta (AIP) and less likely to respond to conservative management [2–4]. The incidence of PAS has been increasing globally in recent years, mainly because cesarean rates have increased to 1:220–533 births worldwide [5–7], with the largest increases found in developing countries [5, 8–10].

PAS is associated with hemorrhagic morbidities such as massive blood loss and massive blood transfusion, which can occur in any degree of PAS severity [11, 12]. Previous studies have found that blood loss of more than 2500 mL was significantly associated with the need for blood transfusion, ICU admission, and other complications associated with bleeding such as acute renal failure or even maternal death [11, 13–15]. Antenatal detection of PAS disorders plays an important role in reducing hemorrhagic morbidities [16–20]. Two-dimensional (2D) ultrasonography is the recommended tool for the diagnosis of PAS, as it has a proven high detection rate and is available in most centers [7, 11, 13, 19, 20]. Accurate prenatal detection of PAS enables timely transfer of care to a tertiary center where appropriate pre-operative readiness of a multidisciplinary team of experienced specialists, intensive care units, blood components and necessary supportive facilities can ensure an optimal outcome [2, 3, 21]. However, there are still some limitations and controversy about the role of antenatal ultrasonography in predicting peri-operative blood loss (PBL) [11, 20, 22, 23]. When the severity of bleeding is underestimated, the preparation of blood components may be inadequate to promptly replace massive bleeding during surgery and can result in several intra- and post-operative complications. Contrarily, if blood loss is overestimated, some of the blood components will be wasted. Therefore, reasonably accurate prediction of intraoperative blood loss is a major concern when preparing for surgery, particularly in developing countries where there might be a shortage of blood donations or blood bank facilities.

In previous studies, a cluster of ultrasound findings suggestive of massive bleeding and co-morbidities associated with PAS disorders have been reported [7, 11, 24–29]. However, the terms used for the diagnosis and different ultrasound signs have been inconsistently reported among these studies. The purpose of this study was to systemically evaluate the two-dimensional ultrasound signs which have been recommended as the main diagnostic modality for PAS as predictive for peri-operative massive hemorrhage in women with placenta previa suspected of PAS.

## Materials and methods

This retrospective study was done in Songklanagarind Hospital, the major tertiary and referral center for prenatal diagnosis and management of PAS in the south of Thailand. The study was approved by the Ethics Committee of the Faculty of Medicine, Prince of Songkla University (#63-663-12-4.). Written informed consent was waived as the data were retrieved retrospectively and anonymized. The inclusion criteria were pregnant women with gestational age of 26–40 weeks with a diagnosis of placenta previa suspicious of PAS disorders such as a history of previous uterine surgery. We reviewed the medical records of 534 pregnant women delivered between January 2007 and May 2021 in our institution. Patients who had incomplete medical records were excluded from the study.

Our prenatal diagnosis protocol used 2D ultrasonography (GE Volusion E8 and S10, GE Medical Systems, Zipf, Austria) performed by maternal-fetal medicine specialists to evaluate women at risk of PAS. The recorded 2D ultrasonographic images used the 2D ultrasound criteria of the European Working Group on Abnormally Invasive Placenta (EW-AIP, which has been changed to IS-PAS (International Society for the Placenta Accreta Spectrum) [30]. Six 2D greyscale and 4 color Doppler ultrasound signs were evaluated in all study patients who were suspected of PAS. All the placenta previa patients suspicious of PAS were prepared and delivered by our PAS team by cesarean section at gestational ages of 34–36 weeks following the protocol of our institution. Following our hospital protocol, the management plan and possible complications were discussed with all patients prior to their surgeries. After the surgery, all hysterectomy cases were pathologically confirmed. Patients in whom the placenta was able to be manually removed during cesarean delivery were diagnosed as placenta previa without PAS.

The study data retrieved from the database system of our institution included patient characteristics, prenatal ultrasound findings, operative notes, delivery outcomes and pathology reports. The primary outcome was peri-operative blood loss (PBL) as recorded in the operative notes. PBL was defined as the summary of blood loss measured from the uterine incision until the end of surgery, as assessed by the contents of the suction canister and blood-soaked materials, less the estimated amount of amniotic fluid collected in the cannister. The secondary outcomes were units of transfused blood components, injury to adjacent organs, ICU admission and post-operative complications. The participants were classified based on PBL into two groups: non-massive (≤ 2500 mL) and massive (>2500 mL) PBL.

The ultrasound findings were evaluated by 2 fetal medicine specialists in our institution. The agreements of each ultrasound sign between the two observers were evaluated 50 cases randomly selected during the study period to which they were blinded from the histopathology results. The agreement between the 2 doctors were between 0.7–1.0 in each ultrasound sign when using Cohen’s Kappa analysis.

The statistical analysis was performed using the R-program. For the demographic data, continuous variables were analyzed as mean or median depending on the distribution of the data. Nominal variables were calculated as absolute number and percent. Odds ratios were calculated for the primary outcome using multivariate analysis. A ROC curve was generated to assess the diagnostic accuracy of the scoring system for the identification of patients who develop massive PBL in PAS. The scoring system was created by using the coefficients of each ultrasound sign and multiplying them into numeric scores. Sensitivities, specificities, false positive rates, false negative rates, positive predictive values and negative predictive values were calculated to create an optimal cut point for the scoring system. Values of P < 0.05 were considered statistically significant.

## Results

**Fig 1:**
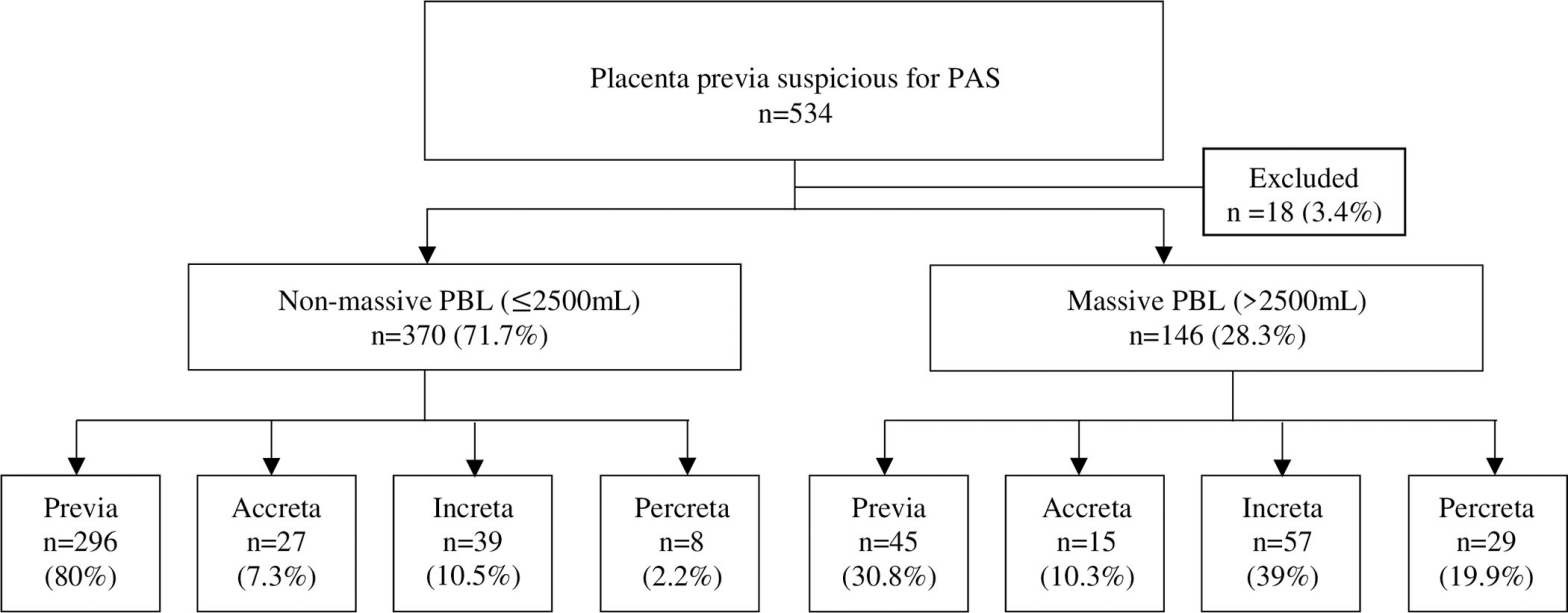
Flow chart of study patients. PAS: placenta accreta spectrum, PBL: perioperative blood loss.

534 pregnant women with a diagnosis of placenta previa suspicious of PAS were identified during the 14-year study period, with 18 patients later excluded due to incomplete records of 2D ultrasound signs or PBL. The remaining 516 patients were classified into 2 groups based on PBL, 370 (71.7%) in the non-massive PBL group and 146 (28.3%) in the massive PBL group as shown in Fig 1. In the non-massive PBL group, most (296, 80%) were placenta previa. In the massive PBL group, the largest group was placenta increta, with 57 patients (39%).

The characteristics between the two groups are compared in Table 1. The medians of maternal age, parity and number of previous cesarean deliveries were all significantly higher in the massive PBL group. The numbers of elective or emergency surgeries were not significantly different between the groups. The median blood loss in the placenta previa group was 1000 mL (IQR 1,533–1,800 mL) and in the PAS group was 3,000 mL (IQR 1,700–3,500 mL). The numbers of patients requiring packed red cells (PRC), fresh frozen plasma (FFP) and platelet concentrations (PC) were all significantly higher in the massive PBL group. The numbers of patients requiring hysterectomy, having intra-operative bladder or ureter injuries, or ICU admission were also higher in the massive PBL group.

**Table 1:** Patient characteristics compared between the non-massive and massive perioperative blood loss (PBL) groups.

	Non-massive PBL	Massive PBL	*P* value
	n (%)	n (%)	
	370 (71.3)	146 (21.1)	
**Age, years (median)**	34	36	0.03
**Weight, kg (median)**	66	65	0.323
**Gravidity (number)**			<0.001
1 (%)	72 (19.5)	11 (7.5)	
≥2 (%)	298 (81.5)	135 (92.5)	
**Parity**			<0.001
0, n (%)	95 (25.7)	13 (8.9)	
≥1, n (%)	275 (74.3)	133 (91.1)	
**Previous cesarean section**			< 0.001
Null, n (%)	186 (50.3)	23 (15.8)	
1, n (%)	124 (33.5)	66 (45.2)	
2, n (%)	45 (12.2)	47 (32.2)	
≥ 3, n (%)	15 (4)	10 (6.9)	
**Emergency/Elective cases**			0.874
Elective cases, n (%)	220 (59.5)	85 (58.2)	
Emergency cases, n (%)	150 (40.5)	61 (41.8)	
**Perioperative blood loss, mL (median)**	1000	4500	<0.001
**Intraoperative transfusion**			
PRC units, (median)	0	5.5 (0, 6)	< 0.001
FFP mL, (median)	0	942.5 (500, 1611)	< 0.001
PC units, (median)	0	0 (0, 6)	< 0.001
**Intraoperative procedures**			
Hysterectomy, n (%)	91 (24.6)	122 (83.6)	< 0.001
Four-vessel ligation, n (%)	14 (3.8)	6 (4.1)	1.0
B-lynch, n (%)	2 (0.5)	1 (0.7)	1.0
**Intraoperative complications**			
Bowel injury, n (%)	1 (0.3)	2 (1.4)	0.194
Bladder injury, n (%)	5 (1.4)	18 (12.3)	< 0.001
Ureter injury, n (%)	0 (0)	5 (3.4)	0.002
**Length of hospital stay, days (median)**	6	10	< 0.001
**ICU admission, n (%)**	18 (4.1)	56 (84.8)	< 0.001

PBL—perioperative blood loss, PRC—packed red cells, FFP—fresh frozen plasma, PC—platelet concentration, ICU—intensive care unit

There were 175 PAS cases out of the total 516 cases, of whom 42 (24%) were placenta accreta, 96 (54.9%) were placenta increta and 37 (21.1%) were placenta percreta, and 341 cases of placenta previa without PAS. The 2D ultrasound signs associated with massive PBL in the PAS group are shown in Table 2, 9 of the 10 having statistical significance. The 2D ultrasound signs that were associated with massive PBL in placenta previa without PAS were loss of clear zone, abnormal placental lacunae and placental lacunae feeder vessels (Table 3).

**Table 2:** Ultrasound signs examined in the study as predictors of massive blood loss in PAS comparing the non-massive PBL and massive PBL groups.

2D Ultrasound sign	Non-massive PBL	Massive PBL	*P* value
	n (%)	n (%)	
	74 (42.3)	101 (57.7)	
Loss of clear zone	61 (82.4)	97 (96)	0.006
Abnormal placental lacunae	55 (74.3)	93 (92.1)	0.003
Bladder wall interruption	40 (54.8)	72 (71.3)	0.037
Myometrial thinning	35 (47.9)	70 (69.3)	0.007
Placental bulge	18 (24.7)	46 (45.5)	0.008
Focal exophytic mass	0 (0)	9 (8.9)	0.011
Uterovesical hypervascularity	55 (75.3)	91 (91)	0.01
Subplacental hypervascularity	15 (20.5)	41 (41)	0.007
Bridging vessels	41 (56.2)	58 (58)	0.932
Placental lacunae feeder vessels	33 (45.2)	72 (72)	<0.001

PAS—placenta accreta spectrum, 2D - two dimensional, PBL—perioperative blood loss

**Table 3:** Ultrasound signs as predictors of massive blood loss in placenta previa without PAS.

2D Ultrasound sign	Non-massive PBL	Massive PBL	*P* value
	n (%)	n (%)	
	296 (86.8)	45 (13.2)	
Loss of clear zone	64 (21.6)	17 (37.8)	0.029
Abnormal placental lacunae	36 (12.2)	12 (26.7)	0.017
Bladder wall interruption,	14 (4.7)	5 (11.1)	0.089
Myometrial thinning	9 (3)	1 (2.2)	1.0
Placental bulge	3 (1)	1 (2.2)	0.434
Focal exophytic mass	1 (0.3)	0 (0)	1.0
Uterovesical hypervascularity	24 (11.4)	8 (21.6)	0.108
Subplacental hypervascularity	6 (2.8)	0 (0)	0.596
Bridging vessels	11 (5.2)	0 (0)	0.378
Placental lacunae feeder vessels	8 (3.8)	5 (13.5)	0.03

2D - two dimensional, PBL—perioperative blood loss

**Fig 2:**
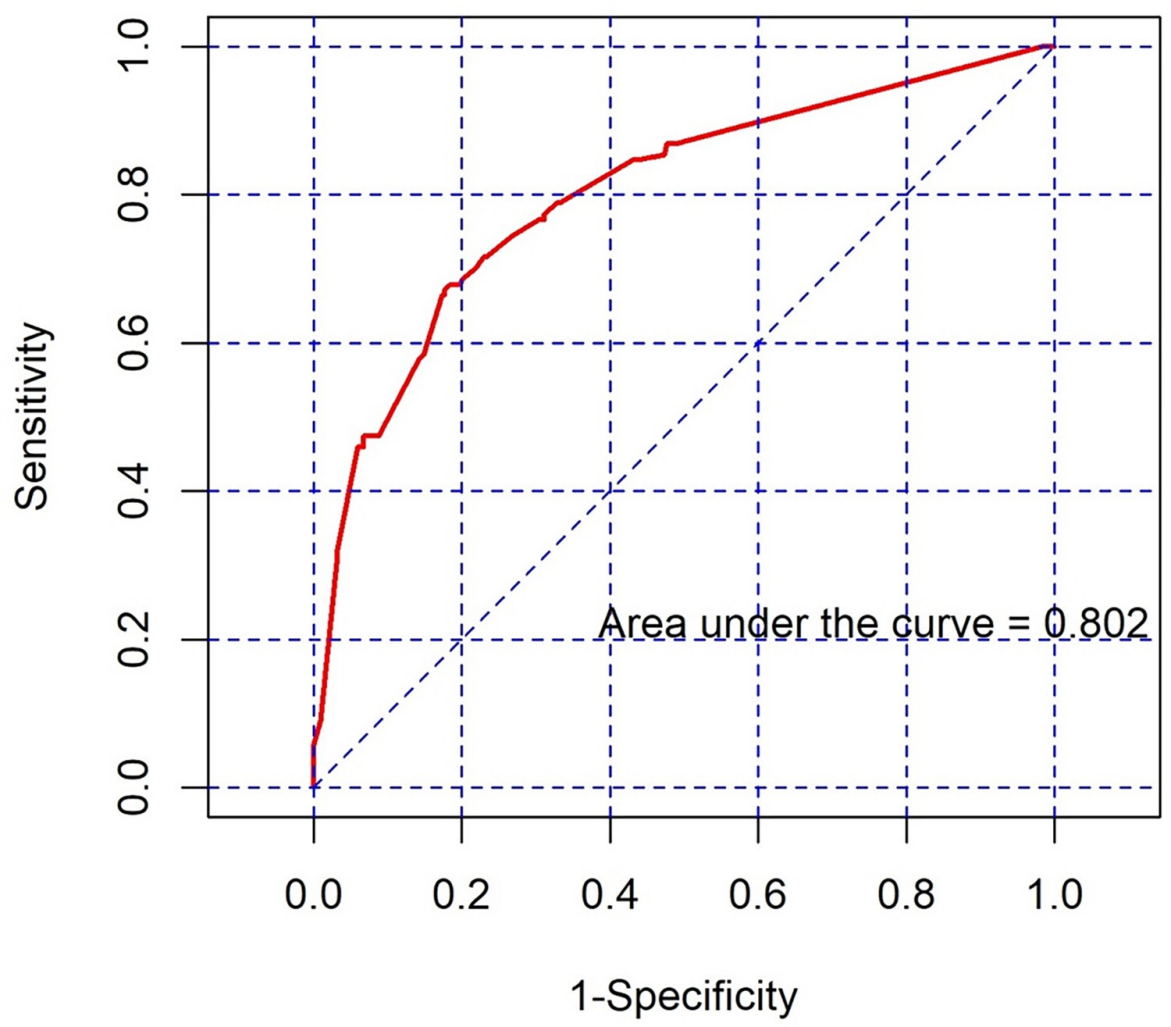
Receiver operator curve of the scoring system to predict massive PBL in PAS disorders. PAS: placenta accreta spectrum, PBL: perioperative blood loss.

The odds ratios (OR) for the different 2D ultrasound signs that were statistically significantly associated with massive PBL in PAS are shown in Table 4. The 3 highest ORs to predict massive PBL were focal exophytic mass, which had an OR of 8.16, placental bulge, which had an OR of 2.47, and placental lacunae feeder with an OR of 2.38. Based on these findings, a scoring system to predict massive PBL in PAS was created by categorizing the signs with the adjusted ORs into a numerical scoring system as shown in Table 5. When using this scoring system to predict massive PBL in PAS, different cutoff scores gave different diagnostic accuracies, as shown in Table 6. The cutoff score of equal to or higher than 2 gave a moderately high sensitivity and positive predictive value and a high specificity and negative predictive value to predict massive PBL in PAS. When using this PAS scoring system, the AUC to predict massive PBL in PAS group was 0.80 (95% CI, 0.76–0.85), as shown in Fig 2.

**Table 4:** Odds ratios of 2D ultrasound signs to predict massive PBL in the PAS group.

2D Ultrasound sign	Crude OR (95%CI)	Adjusted OR (95%CI)	*P* value
Loss of clear zone	6.24 (3.83,10.17)	1.96 (1.01,3.78)	0.047
Abnormal placental lacunae	6.94 (4.37,11.03)	1.86 (0.93,3.72)	0.083
Placental bulge	6.52 (3.69,11.49)	2.47 (1.23,4.97)	0.01
Focal exophytic mass	19.83 (2.49,158.13)	8.16 (0.89,74.47)	0.024
Uterovesical hypervascularity	6.85 (4.34,10.8)	2.19 (1.07,4.47)	0.033
Bridging vessels	3.26 (2.07,5.13)	0.47 (0.24,0.91)	0.022
Placental lacunae feeder vessels	7.8 (4.85,12.54)	2.38 (1.23,4.6)	0.009

2D - two dimensional, PBL—perioperative blood loss, OR—odds ratio, PAS—placenta accreta spectrum, CI -confidence interval

**Table 5:** Scores for each ultrasound sign to predict massive PBL in the PAS group.

2D Ultrasound sign	Score
Loss of clear zone	0.5
Abnormal placental lacunae	0.5
Placental bulge	1.0
Focal exophytic mass	2.0
Uterovesical hypervascularity	1.0
Placental lacunae feeder vessels	1.0
Bridging vessels	-1.0

PBL—perioperative blood loss, PAS—placenta accreta spectrum, 2D - two dimensional

**Table 6:** Diagnostic accuracy of various cut points of the PAS scoring system to predict massive PBL.

Cut point of score	Sensitivity	Specificity	FPR	FNR	PPV	NPV
≥ 5.0	4%	100%	0%	95%	100%	68%
≥ 4.0	9%	99%	1%	91%	81%	69%
≥ 3.5	10%	99%	1%	90%	82%	69%
≥ 3.0	46%	94%	6%	54%	79%	78%
≥ 2.5	47%	93%	7%	53%	77%	79%
≥ 2.0	66%	82%	18%	34%	65%	84%
≥ 1.5	72%	77%	23%	28%	60%	85%
≥ 1.0	78%	67%	33%	22%	54%	86%
≥ 0.5	85%	53%	47%	15%	47%	88%

PAS—placenta accreta spectrum, PBL—perioperative blood loss, FPR—false positive rate, FNR—false negative rate, PPV—positive predictive value, NPV—negative predictive value

## Discussion

Massive bleeding is one of the most common problems in the management of PAS whether the patient is having conservative surgery or a cesarean hysterectomy [19, 31, 32]. The average blood loss during PAS surgery has been reported as varying between 2000 and 5000 mL, levels which require in turn massive blood transfusion [13, 16, 33, 34]. PAS also involves increased rates of organ injuries and ICU admissions and longer hospital stays, as found in our study and other studies [11, 13, 35, 36]. Our study was done in the main tertiary referral center for managing PAS in the south of Thailand, where the incidence of PAS has been increasing for several years now and recently reported the very high rate of 1:161 deliveries [10]. The average blood loss in PAS during the study period was 2500 mL, which is in the same range as other studies [34–37]. In our study, most of the PAS cases underwent a cesarean hysterectomy (94%) without trying to remove the placenta as the preferred surgical technique to prevent massive blood loss, as has been reported in other studies [2, 21, 36, 38]. PBL amounts of more than 2500 mL have been associated with significant hemorrhagic morbidities [34, 35, 39]. Accurate estimation of the expected blood loss during an operation and preparation of the appropriate amount of blood products for transfusion is one of the major concerns when preparing for PAS surgeries, particularly in non-tertiary hospitals or hospitals with no blood bank [2, 19, 39]. If enough blood components are not prepared or available, the patient may face the morbidity of massive hemorrhage or even death, [14, 15] while if an unnecessarily large amount is prepared, there will be wastage, which is especially to be avoided in areas that lack a blood bank [40, 41].

Various methods to predict blood loss in PAS have been reported in previous studies, including the use of pregnancy histories, various ultrasound signs with 2D and/or 3D techniques or even MRI [7, 11–13, 24, 26, 27, 34, 35], although MRI has shown low accuracy in predicting severe PAS disorders [29]. In our institution, we used 2D ultrasound with the EW-AIP criteria to diagnose PAS, which provides detailed descriptions of various ultrasound signs and have been accepted worldwide, including during the study period [11, 13, 30, 42]. In our study, 9 of the 10 signs described by the EW-AIP criteria were significantly associated with massive PBL of >2500 mL in the PAS group. Only the sign of bridging vessels was not associated with massive PBL, but did show a protective effect, a finding consistent with some previous studies [11, 13, 28] which did not include bridging vessels in their scoring systems to predict the severity of PAS. However, some other studies have reported an association of bridging vessels with massive PBL [7, 24]. Based on our analysis (Table 3), we recommend that if at least 1 significant ultrasound sign is found in a case of placenta previa without PAS, blood components should be prepared for transfusion and the surgical team alerted because of an increased risk of postpartum hemorrhage and the possible need of blood transfusion.

When comparing the amount of PAS blood loss in various studies, the differences in blood loss may involve many factors such as prenatal diagnosis, grade of PAS, management strategy of conservative treatment or cesarean hysterectomy, experience of the operator, gestational age of delivery, and whether elective or emergency surgery [11, 29, 35]. In our study, all placenta previa suspicious of PAS cases were antenatally diagnosed. There were 133 (84.2%) AIP cases in our study, which are generally less likely to be treated with conservative surgery, [19, 29, 42] and all of these patients were counseled and recommended to have elective cesarean hysterectomies between 34–36 weeks following the ACOG recommendations [19].

During the 14-year study period, we had a few staff changes due to retirement. But the most important thing that changed was the standard surgical technique for PAS cases in our institution. We modified our surgical technique to reduce blood loss in PAS cases by using a midline incision of the uterus to avoid placental bleeding, superior devascularization of utero-ovarian pedicles bilaterally with bipolar vascular sealing, a retroperitoneal dissection to ligate the anterior branch of the internal iliac arteries and a colpotomy with a posterior approach technique. So, these changes may have affected the reduction of overall blood loss in our institution. In terms of delivery settings, the rates of elective and emergency surgery were not different between the massive and non-massive PBL groups.

Our new scoring system provides a highly accurate and simple model for predicting massive PBL with an AUC of 0.80 (95% CI, 0.76–0.85). A previous multicenter study by Zheng et al. [7] showed an AUC of 0.76 to predict massive PBL ≥ 1500 mL and another by Shazly et al. [35] used a machine learning model to predict massive PBL ≥ 2500 mL with an AUC of 0.84, but both systems were based on complex formulas which are difficult for practical use and not generalizable. These 2 studies included large numbers of PAS cases but the Zheng study included only 67 AIP cases (3.1% of all cases) to verify the scoring system [7], and also the variety of management strategies for PAS cases inevitable in multicenter studies could affect the outcomes [29, 35]. Our study did not use maternal history in the scoring system as in some other studies [7, 26, 28, 35] because a history of previous surgery and/or placenta previa were found in all cases of PAS reported in previous studies [24, 26, 27]. In our study, we created a scoring system focusing on the hemorrhagic outcomes more than the grade of invasion because this is more related to clinical outcomes. However, some studies have given more attention to PAS grading [26, 27, 29]. For example, the study of Gilbo et al. [27] reported using only three 2D ultrasound signs for their scoring system. However, at least 2 of their signs are always found in any degree of PAS, which may not directly relate to clinical outcomes. The study of Morel et al. [29] included a large numbers of PAS cases without a definite predicting system, but did not find any correlations between antenatal ultrasound signs and grade of PAS. The scoring system of Tovbin et al. [24] focused on the probability of a PAS diagnosis but was not related to clinical outcomes. The study of Cali G et al. [11] showed correlations between a group of ultrasound findings and the FIGO 2018 clinical grading system which were related to clinical outcomes, however, the hemorrhagic outcomes of this study were much lower than in previous studies, [10, 12, 28, 34, 35] even in the most severe group, which may reflect different management protocols among the centers.

In Table 6, we show the different cutoff scores of our scoring system to predict massive PBL in PAS. We propose that two different cut offs would be useful depending on two common different clinical scenarios. For primary and secondary care hospitals that lack specialists or a blood bank, the cutoff score of ≥ 1.5 should be used as the referral point, while for tertiary hospitals that have the necessary facilities and multidisciplinary teams to properly manage PAS, a cutoff ≥ 2 is appropriate as an indicator to prepare blood products for transfusion, prepare for a hysterectomy or prepare for adjuvant hemostatic procedures or intervention radiology to stop bleeding, which is still not a routine recommendation in most hospitals [12, 35]. Using this system can help obstetricians to manage PAS cases more confidently, especially in hospitals that lack basic facilities, or assess when it is indicated to refer a patient to a tertiary center in a timely manner to reduce morbidity and mortality from postpartum hemorrhage, which remains a core problem in obstetrics, particularly during this period of increasing rates of cesarean deliveries worldwide.

The main strengths of our study were that our data were based on placenta previa suspected of PAS cases in a single center and we had a large sample size and a high number of AIP cases. Also, we used a standard management protocol of PAS in our study during the study period implemented by the multidisciplinary PAS care team of our institution from diagnosis to delivery, unlike multicenter studies which would have different management methods. Our scoring system is based on the 2D ultrasound technique which is available in all hospitals, is not expensive and does not need extra training as with 3D ultrasound or MRI. Also, our institution reported high accuracies in diagnosing PAS of between 91 and 94% [10], similar to other PAS centers [43, 44]. In our center, we have a dedicated PAS surgical care team in which all surgeons are gynecologic oncologists with many years of experience in difficult surgical conditions such as PAS, so the average amount of blood loss in our study may have been less than in studies from other centers which may have been influenced by factors such as surgeon experience, surgical technique, accuracy of prenatal diagnosis, etc. [7, 12]. All of our cases of massive PBL subsequently diagnosed as PAS had the diagnosis confirmed by histology, which is still the gold standard to diagnose this condition. A recent study by Ishibashi et al. also found that the severity of bleeding was correlated to PAS histology grading [12]. In the placenta previa only group, our study adds some new information on using ultrasound signs to predict massive PBL in this group that will be of benefit to other obstetricians in the preparation of a management care team and blood components for cases of placenta previa which are considered at risk of massive hemorrhage.

There were also some limitations to this study, primarily due to its retrospective design. Our study did not evaluate possible correlations between the ultrasound signs and the FIGO 2018 clinical grades [45] as most of our cases underwent surgery before the FIGO grading system was implemented. Also, the number of focal lesions or placenta accreta cases which had successful conservative surgery may have been underestimated.

## Conclusion

Predicting massive PBL hemorrhage by using our new scoring system based on the common 2D grey scale and color Doppler ultrasound signs is easy to do and can be useful in helping to optimize pre-operative management in cases of placenta previa suspicious of PAS. However, further prospective studies are needed to validate the scoring system.

## Supporting information

S1 Dataset (CSV)

S1 Table Table of agreement of ultrasound signs between the 2 sonographers. (DOCX)

## References

[1] Jauniaux E, Jurkovic D. Placenta accreta: pathogenesis of a 20th century iatrogenic uterine disease. Placenta 2012; 33: 244–251. doi: 10.1016/j.placenta.2011.11.010 22284667

[2] Allen L, Jauniaux E, Hobson S, Papillon-Smith J, Belfort MA. FIGO consensus guidelines on placenta accreta spectrum disorders: Nonconservative surgical management. Int J Gynaecol Obstet 2018; 140: 281–290. doi: 10.1002/ijgo.12409 29405317

[3] Sentilhes L, Kayem G, Chandraharan E, Palacios-Jaraquemada J, Jauniaux E. FIGO consensus guidelines on placenta accreta spectrum disorders: Conservative management. Int J Gynaecol Obstet 2018; 140: 291–298. doi: 10.1002/ijgo.12410 29405320

[4] Morlando M, Collins S. Placenta Accreta Spectrum Disorders: Challenges, Risks, and Management Strategies. Int J Womens Health 2020; 12: 1033–1045. doi: 10.2147/IJWH.S224191 33204176 PMC7667500

[5] Wu S, Kocherginsky M, Hibbard JU. Abnormal placentation: twenty-year analysis. Am J Obstet Gynecol 2005; 192: 1458–1461. doi: 10.1016/j.ajog.2004.12.074 15902137

[6] Jauniaux E, Ayres-de-Campos D. FIGO consensus guidelines on placenta accreta spectrum disorders: Introduction. Int J Gynaecol Obstet 2018; 140: 261–264. doi: 10.1002/ijgo.12406 29405322

[7] Zheng W, Zhang H, Ma J, Dou R, Zhao X, Yan J, et al. Validation of a scoring system for prediction of obstetric complications in placenta accreta spectrum disorders. J Matern Fetal Neonatal Med 2021. doi: 10.1080/14767058.2020.1847077 1–7. 33685330

[8] Jauniaux E, Chantraine F, Silver RM, Langhoff-Roos J. FIGO consensus guidelines on placenta accreta spectrum disorders: Epidemiology. Int J Gynaecol Obstet 2018; 140: 265–273. doi: 10.1002/ijgo.12407 29405321

[9] Morlando M, Sarno L, Napolitano R, Capone A, Tessitore G, Maruotti GM, et al. Placenta accreta: incidence and risk factors in an area with a particularly high rate of cesarean section. Acta Obstet Gynecol Scand 2013; 92: 457–460. doi: 10.1111/aogs.12080 23347183

[10] Maison N, Rattanaburi A, Pruksanusak N, Buhachat R, Tocharoenvanich S, Harnprasertpong J, et al. Intraoperative blood volume loss according to gestational age at delivery among pregnant women with placenta accreta spectrum (PAS): an 11-year experience in Songklanagarind Hospital. J Obstet Gynaecol 2021. doi: 10.1080/01443615.2021.1910638 1–6. 34155959

[11] Cali G, Forlani F, Lees C, Timor-Tritsch I, Palacios-Jaraquemada J, Dall’Asta A, et al. Prenatal ultrasound staging system for placenta accreta spectrum disorders. Ultrasound Obstet Gynecol 2019; 53: 752–760. doi: 10.1002/uog.20246 30834661

[12] Ishibashi H, Miyamoto M, Iwahashi H, Matsuura H, Kakimoto S, Sakamoto T, et al. Criteria for placenta accreta spectrum in the International Federation of Gynaecology and Obstetrics classification, and topographic invasion area are associated with massive hemorrhage in patients with placenta previa. Acta Obstet Gynecol Scand 2021; 100: 1019–1025. doi: 10.1111/aogs.14143 33715171

[13] Dall’Asta A, Calì G, Forlani F, Paramasivam G, Girardelli S, Yazbek J, et al. Evaluation of perioperative complications using a newly described staging system for placenta accreta spectrum. Eur J Obstet Gynecol Reprod Biol 2020; 250: 54–60. doi: 10.1016/j.ejogrb.2020.04.038 32387893

[14] Shamshirsaz AA, Fox KA, Salmanian B, Diaz-Arrastia CR, Lee W, Baker BW, et al. Maternal morbidity in patients with morbidly adherent placenta treated with and without a standardized multidisciplinary approach. Am J Obstet Gynecol 2015; 212: 218.e211–219. doi: 10.1016/j.ajog.2014.08.019 25173187

[15] Lal AK, Hibbard JU. Placenta previa: an outcome-based cohort study in a contemporary obstetric population. Arch Gynecol Obstet 2015; 292: 299–305. doi: 10.1007/s00404-015-3628-y 25638449

[16] Shamshirsaz AA, Fox KA, Erfani H, Clark SL, Shamshirsaz AA, Nassr AA, et al. Outcomes of Planned Compared With Urgent Deliveries Using a Multidisciplinary Team Approach for Morbidly Adherent Placenta. Obstet Gynecol 2018; 131: 234–241. doi: 10.1097/AOG.0000000000002442 29324609

[17] Bartels HC, Rogers AC, O’Brien D, McVey R, Walsh J, Brennan DJ. Association of Implementing a Multidisciplinary Team Approach in the Management of Morbidly Adherent Placenta With Maternal Morbidity and Mortality. Obstet Gynecol 2018; 132: 1167–1176. doi: 10.1097/AOG.0000000000002865 30234729

[18] Del Negro V, Aleksa N, Galli C, Ciminello E, Derme M, Vena F, et al. Ultrasonographic Diagnosis of Placenta Accreta Spectrum (PAS) Disorder: Ideation of an Ultrasonographic Score and Correlation with Surgical and Neonatal Outcomes. Diagnostics (Basel) 2020; 11. doi: 10.3390/diagnostics11010023 33375532 PMC7824485

[19] Obstetric Care Consensus No. 7: Placenta Accreta Spectrum. Obstet Gynecol 2018; 132: e259–e275. doi: 10.1097/AOG.0000000000002983 30461695

[20] Jauniaux E, Bhide A, Kennedy A, Woodward P, Hubinont C, Collins S. FIGO consensus guidelines on placenta accreta spectrum disorders: Prenatal diagnosis and screening. Int J Gynaecol Obstet 2018; 140: 274–280. doi: 10.1002/ijgo.12408 29405319

[21] Jauniaux E, Alfirevic Z, Bhide AG, Belfort MA, Burton GJ, Collins SL, et al. Placenta Praevia and Placenta Accreta: Diagnosis and Management: Green-top Guideline No. 27a. Bjog 2019; 126: e1–e48. doi: 10.1111/1471-0528.15306 30260097

[22] Jauniaux E, Collins SL, Jurkovic D, Burton GJ. Accreta placentation: a systematic review of prenatal ultrasound imaging and grading of villous invasiveness. Am J Obstet Gynecol 2016; 215: 712–721. doi: 10.1016/j.ajog.2016.07.044 27473003

[23] D’Antonio F, Iacovella C, Bhide A. Prenatal identification of invasive placentation using ultrasound: systematic review and meta-analysis. Ultrasound Obstet Gynecol 2013; 42: 509–517. doi: 10.1002/uog.13194 23943408

[24] Tovbin J, Melcer Y, Shor S, Pekar-Zlotin M, Mendlovic S, Svirsky R, et al. Prediction of morbidly adherent placenta using a scoring system. Ultrasound Obstet Gynecol 2016; 48: 504–510. doi: 10.1002/uog.15813 26574157

[25] Hussein AM, Elbarmelgy RA, Elbarmelgy RM, Thabet MM, Jauniaux E. Prospective evaluation of the impact of post-cesarean section uterine scarification in the perinatal diagnosis of placenta accreta spectrum. Ultrasound Obstet Gynecol 2021. doi: 10.1002/uog.23732 34225385 PMC9311077

[26] Rac MW, Dashe JS, Wells CE, Moschos E, McIntire DD, Twickler DM. Ultrasound predictors of placental invasion: the Placenta Accreta Index. Am J Obstet Gynecol 2015; 212: 343.e341–347. doi: 10.1016/j.ajog.2014.10.022 25446658

[27] Gilboa Y, Spira M, Mazaki-Tovi S, Schiff E, Sivan E, Achiron R. A novel sonographic scoring system for antenatal risk assessment of obstetric complications in suspected morbidly adherent placenta. J Ultrasound Med 2015; 34: 561–567. doi: 10.7863/ultra.34.4.561 25792570

[28] Chong Y, Zhang A, Wang Y, Chen Y, Zhao Y. An ultrasonic scoring system to predict the prognosis of placenta accreta: A prospective cohort study. Medicine (Baltimore) 2018; 97: e12111. doi: 10.1097/MD.0000000000012111 30170439 PMC6392640

[29] Morel O, van Beekhuizen HJ, Braun T, Collins S, Pateisky P, Calda P, et al. Performance of antenatal imaging to predict placenta accreta spectrum degree of severity. Acta Obstet Gynecol Scand 2021; 100 Suppl 1: 21–28. doi: 10.1111/aogs.14112 33811333 PMC8252006

[30] Collins SL, Ashcroft A, Braun T, Calda P, Langhoff-Roos J, Morel O, et al. Proposal for standardized ultrasound descriptors of abnormally invasive placenta (AIP). Ultrasound Obstet Gynecol 2016; 47: 271–275. doi: 10.1002/uog.14952 26205041

[31] Creanga AA, Bateman BT, Butwick AJ, Raleigh L, Maeda A, Kuklina E, et al. Morbidity associated with cesarean delivery in the United States: is placenta accreta an increasingly important contributor? Am J Obstet Gynecol 2015; 213: 384.e381–311. doi: 10.1016/j.ajog.2015.05.002 25957019

[32] Collins SL, Alemdar B, van Beekhuizen HJ, Bertholdt C, Braun T, Calda P, et al. Evidence-based guidelines for the management of abnormally invasive placenta: recommendations from the International Society for Abnormally Invasive Placenta. Am J Obstet Gynecol 2019; 220: 511–526. doi: 10.1016/j.ajog.2019.02.054 30849356

[33] Shazly SA, Hortu I, Shih JC, Melekoglu R, Fan S, Ahmed FUA, et al. Prediction of clinical outcomes in women with placenta accreta spectrum using machine learning models: an international multicenter study. J Matern Fetal Neonatal Med 2021. doi: 10.1080/14767058.2021.1918670 1–10. 34233555

[34] Hussein AM, Momtaz M, Elsheikhah A, Abdelbar A, Kamel A. The role of ultrasound in prediction of intra-operative blood loss in cases of placenta accreta spectrum disorders. Arch Gynecol Obstet 2020; 302: 1143–1150. doi: 10.1007/s00404-020-05707-y 32740869

[35] Shazly SA, Hortu I, Shih JC, Melekoglu R, Fan S, Ahmed FUA, et al. Prediction of success of uterus-preserving management in women with placenta accreta spectrum (CON-PAS score): A multicenter international study. Int J Gynaecol Obstet 2021; 154: 304–311. doi: 10.1002/ijgo.13518 33278833

[36] Morlando M, Schwickert A, Stefanovic V, Gziri MM, Pateisky P, Chalubinski KM, et al. Maternal and neonatal outcomes in planned versus emergency cesarean delivery for placenta accreta spectrum: A multinational database study. Acta Obstet Gynecol Scand 2021; 100 Suppl 1: 41–49. doi: 10.1111/aogs.14120 33713033

[37] Bloomfield V, Rogers S, Scattolon S, Morais M, Leyland N. Informing the Spectrum of Approaches: Institutional Review of Placenta Accreta Spectrum Disorders Management. J Obstet Gynaecol Can 2021. doi: 10.1016/j.jogc.2021.10.013 34740850

[38] Cahill AG, Beigi R, Heine RP, Silver RM, Wax JR. Placenta Accreta Spectrum. Am J Obstet Gynecol 2018; 219: B2–b16. 10.1016/j.ajog.2018.09.042 30471891

[39] Guasch E, Gilsanz F. Massive obstetric hemorrhage: Current approach to management. Med Intensiva 2016; 40: 298–310. doi: 10.1016/j.medin.2016.02.010 27184441

[40] Bharat S, Rahul K, Indraneel D, Anubha S, Atul P, Preeti E, et al. Impact of COVID-19 pandemic on the pattern of blood donation and blood safety: Experience from a hospital-based blood center in North India. Asian J Transfus Sci 2021; 15: 119–124. doi: 10.4103/ajts.ajts_29_21 34908741 PMC8628224

[41] Kandasamy D, Shastry S, Chenna D, Mohan G. COVID-19 pandemic and blood transfusion services: The impact, response and preparedness experience of a tertiary care Blood Center in southern Karnataka, India. Hematol Transfus Cell Ther 2021. doi: 10.1016/j.htct.2021.09.019 34931179 PMC8674497

[42] Schwickert A, van Beekhuizen HJ, Bertholdt C, Fox KA, Kayem G, Morel O, et al. Association of peripartum management and high maternal blood loss at cesarean delivery for placenta accreta spectrum (PAS): A multinational database study. Acta Obstet Gynecol Scand 2021; 100 Suppl 1: 29–40. doi: 10.1111/aogs.14103 33524163

[43] Jauniaux E, Bhide A. Prenatal ultrasound diagnosis and outcome of placenta previa accreta after cesarean delivery: a systematic review and meta-analysis. Am J Obstet Gynecol 2017; 217: 27–36. doi: 10.1016/j.ajog.2017.02.050 28268196

[44] Bailit JL, Grobman WA, Rice MM, Reddy UM, Wapner RJ, Varner MW, et al. Morbidly adherent placenta treatments and outcomes. Obstet Gynecol 2015; 125: 683–689. doi: 10.1097/AOG.0000000000000680 25730233 PMC4347990

[45] Jauniaux E, Ayres-de-Campos D, Langhoff-Roos J, Fox KA, Collins S. FIGO classification for the clinical diagnosis of placenta accreta spectrum disorders. Int J Gynaecol Obstet 2019; 146: 20–24. doi: 10.1002/ijgo.12761 31173360

