# Supplementary material for: Two-dimensional ultrasound signs as predictive markers of massive peri-operative blood loss in placenta previa suspicious for placenta accreta spectrum (PAS) disorder
Source: PLoS One. 2022 Oct 14;17(10):e0276153. doi: 10.1371/journal.pone.0276153 (PMC9565412; doi:10.1371/journal.pone.0276153)
Supplement: S1 Table — (DOCX) [file pone.0276153.s002.docx]

Supplement Table 1 Table of agreement of ultrasound signs between the 2 sonographers

| **Ultrasound signs** | **Kappa values** |
| --- | --- |
| Loss of clear zone | 0.96 |
| Abnormal placental lacunae | 0.87 |
| Bladder wall interruption | 0.87 |
| Myometrial thinning | 0.96 |
| Placental bulge | 1.00 |
| Focal exophytic mass | 0.70 |
| Uterovesical hypervascularity | 0.92 |
| Subplacental hypervascularity | 0.93 |
| Bridging vessels | 0.73 |
| Placental lacunae feeder vessels | 0.89 |
